# Supplementary material for: Distinguishing mechanisms of social contagion from local network view
Source: Npj Complex. 2025 Mar 4;2(1):8. doi: 10.1038/s44260-025-00034-2 (PMC11879858; doi:10.1038/s44260-025-00034-2)
Supplement: Supplementary file 1 — Supplementary Material [file 44260_2025_34_MOESM1_ESM.pdf]

Supplementary Material

Distinguishing mechanisms of social contagion from local network view

1 Contagion curves of the extreme values of the phase space

We investigate the speed of spreading in processes governed by the simple and the complex contagion mechanisms independently. These two dynamical processes are implemented separately on an Erdős–Rényi network of 1000 nodes, with an average degree of 4. We examine the speed of spreading as the function of the simple and complex spreading parameters of β or ϕ (respectively), taking values from a broad range between 0 and 1 (cf Figure S1). High values of β characterise simple contagion processes with high speed since nodes in this scenario have a higher probability to be infected, commonly after a single stimulus. The opposite effect characterise complex contagion: if ϕ is high, the propagation is slow-downed as the proportion of infected neighbours needed adoption is large.

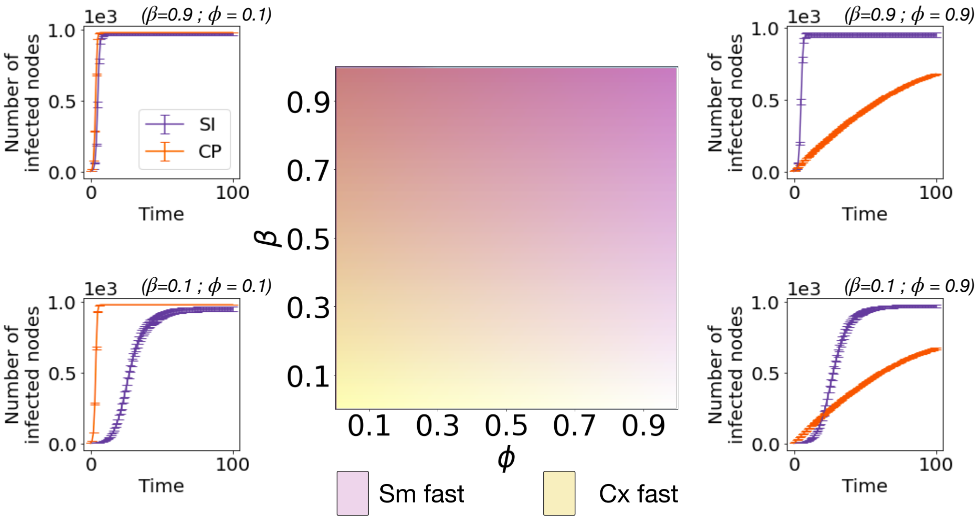

Figure S1. (central panel) Speed dependency of the simple and complex contagions on the epidemic parameters β and ϕ (respectively). The purple and orange colours display respectively the areas where the simple and the complex contagions are faster and are schematic. The number of infected nodes through time is shown on the four extremes of the parameter space, in purple for the simple contagion and in orange for the complex contagion.

2 Selection of the algorithm of machine learning for the classification in Experiment 2

We investigate to find the best classification machine learning algorithm to distinguish between the simple, complex and spontaneous contagions in Experiment 2. We test the following 9 algorithms:

- *Naives Bayes*[1]*:* algorithm which classifies instances using the Bayes' theorem under the hypothesis that every pair of features are independent.
- *K-nearest neighbors (Knn)*[2]*:* the training instances are displayed in a space of the dimension of the number of features. When classifying a contagion case, it is assigned to the same category as the majority of its closest neighbors in this feature space.
- *Perceptron*[3]*:* classifier that learns by iteratively adjusting weights. It utilizes a threshold function to determine the class of the instances based on the dot product of input features and learned weights.

- *Support Vector Classification (SVM)*[4]*:* algorithm that identifies an optimal hyperplane to separate data into different classes by maximizing the margin between the classes.
- *Linear Support Vector Classification (Linear SVM)*[4]*:* variant of Support Vector Classification that specifically employs a linear decision boundary to classify data points into distinct categories.
- *Decision tree*[5]*:* algorithm that recursively partitions data based on feature attributes to construct a hierarchical tree structure for classification
- *Random forest*[6]*:* method which builds multiple decision trees during training and combines their predictions through averaging their results.
- *Ada boost*[7]*:* boosting algorithm that sequentially trains weak learners by emphasizing misclassified instances in subsequent iterations, and thus build a strong classifier by combining the predictions of these weak learners.
- *Gradient boosting*[8]*:* boosting algorithm that sequentially trains weak learners giving more weight to the misclassified instances based on gradients of a loss function.

We present the mean accuracies over the whole parameter-space in the classification of the instances from Experiment 2 for each machine learning algorithm in Table S1. Among the algorithms displaying the highest accuracies (above 0.82), we opt for the *Random forest* method first due to its significantly faster computation times compared to *SVM*. Additionally, the *Random forest* algorithm, consisting of an ensemble of decision trees whose outcomes are combined, generally outperforms individual *Decision Tree* methods. Finally, we exclude the Gradient Boosting algorithm due to its limited explainability.

Naives Bayes	Knn	Perceptron	Linear SVM	SVM	Decision tree	Random forest	Ada boost	Gradient boosting
0.66	0.81	0.68	0.81	0.82	0.82	0.82	0.75	0.83

Table S1. Average over the whole parameter-space of the accuracies on the classification of the contagion cases from Experiment 2

3 Distribution of the features of the random forest of Experiment 2

The features of the random forest have been chosen to present different values according to the mechanisms of adoption. As depicted in Figure S2, the distributions of most features differ for the simple, complex and spontaneous adoptions. The degree is the only feature which is not related to the propagation itself but to the structure of the network. While it does not present significant differences in the distributions within Erdős–Rényi networks, we keep it due to the potential influence of a node's degree in other type of networks.

4 Best subset of features for the random forest on Experiment 2

To evaluate the significance of the features of the random forest method on Experiment 2 and 3, we train algorithms with all possible subsets of the eight features. Figures S3 and S4 present the subset with the highest accuracy (y-axis) for each subset length (x-axis), respectively for Experiments 2 and 3, across the all parameter-space, with corresponding accuracy values indicated in blue. Looking at the results from Experiment 2, enlarging the feature set from one to three increases the accuracy, but a plateau is reached for subsets larger than four. In other words, in most regions of the parameter space, only three features are necessary to achieve the same accuracy as with more features. However, this optimal subset varies through the parameter space. Also, adding features increases the accuracy when ϕ is high, but does not have any effect when the value of ϕ is small. Differently, the set of feature for the classification of Experiment 3 does not have a great influence on the accuracies, as the obtained values for different length of subsets are very similar. In contrast, the selection of features for the classification in Experiment 3 has a limited influence on the accuracies, as the obtained values for different subset lengths are very similar.

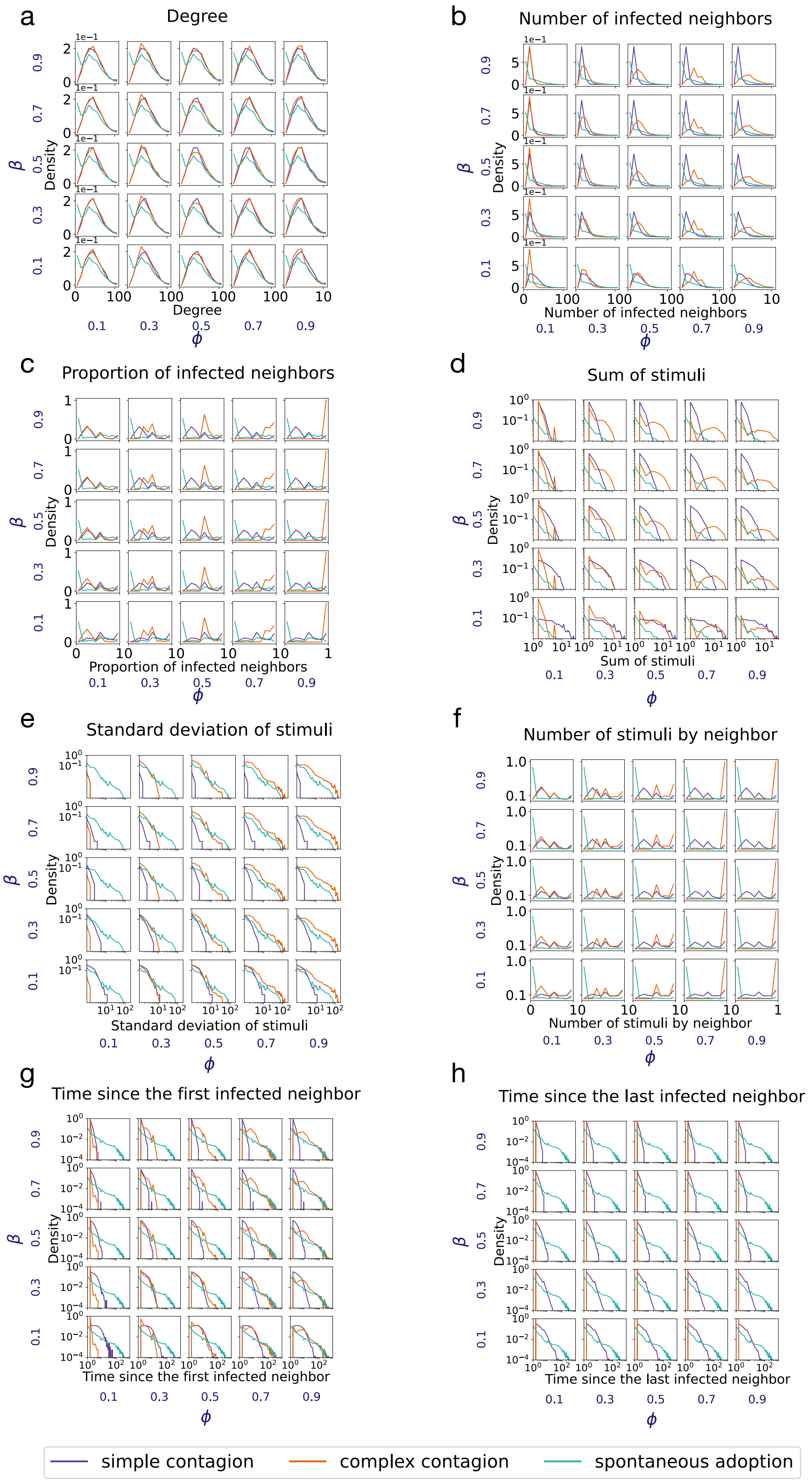

Figure S2. Distribution of the features of the random forest algorithms across the parameter space : degree (panel a), number of infected neighbours (panel b), proportion of infected neighbours (panel c), sum of stimuli (panel d), standard deviation of stimuli (panel e), number of stimuli by neighbours (panel f), time since the first infected neighbour (panel g) and time since the last infected neighbour (panel h). The displayed values, taken from Experiment 2, are grouped by their dynamical processes (simple, complex or spontaneous).

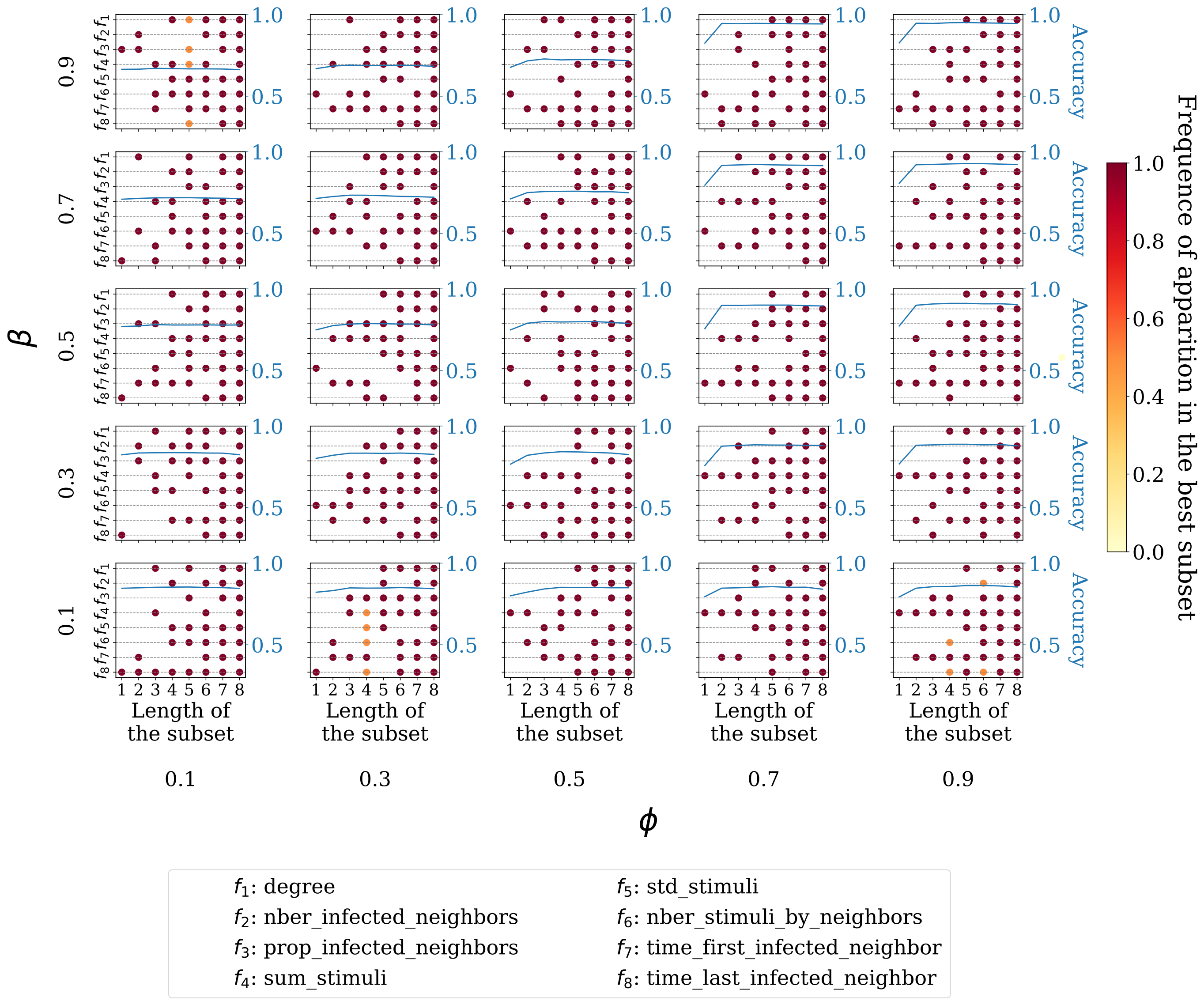

Figure S3. Subsets of features giving the best accuracies (y-axis of each subplot) in the parameter space (β, ϕ) for a certain length of subset (x-axis of each subplot) in the classification with the random forest of Experiment 2. The corresponding accuracies are displayed in blue. If several subsets give the same best accuracies, we compute the frequency of apparition of each feature in those subsets. In most of the cases, only three features is enough to obtain the same accuracy values than with the total set of features, but those three features are different across the parameter space.

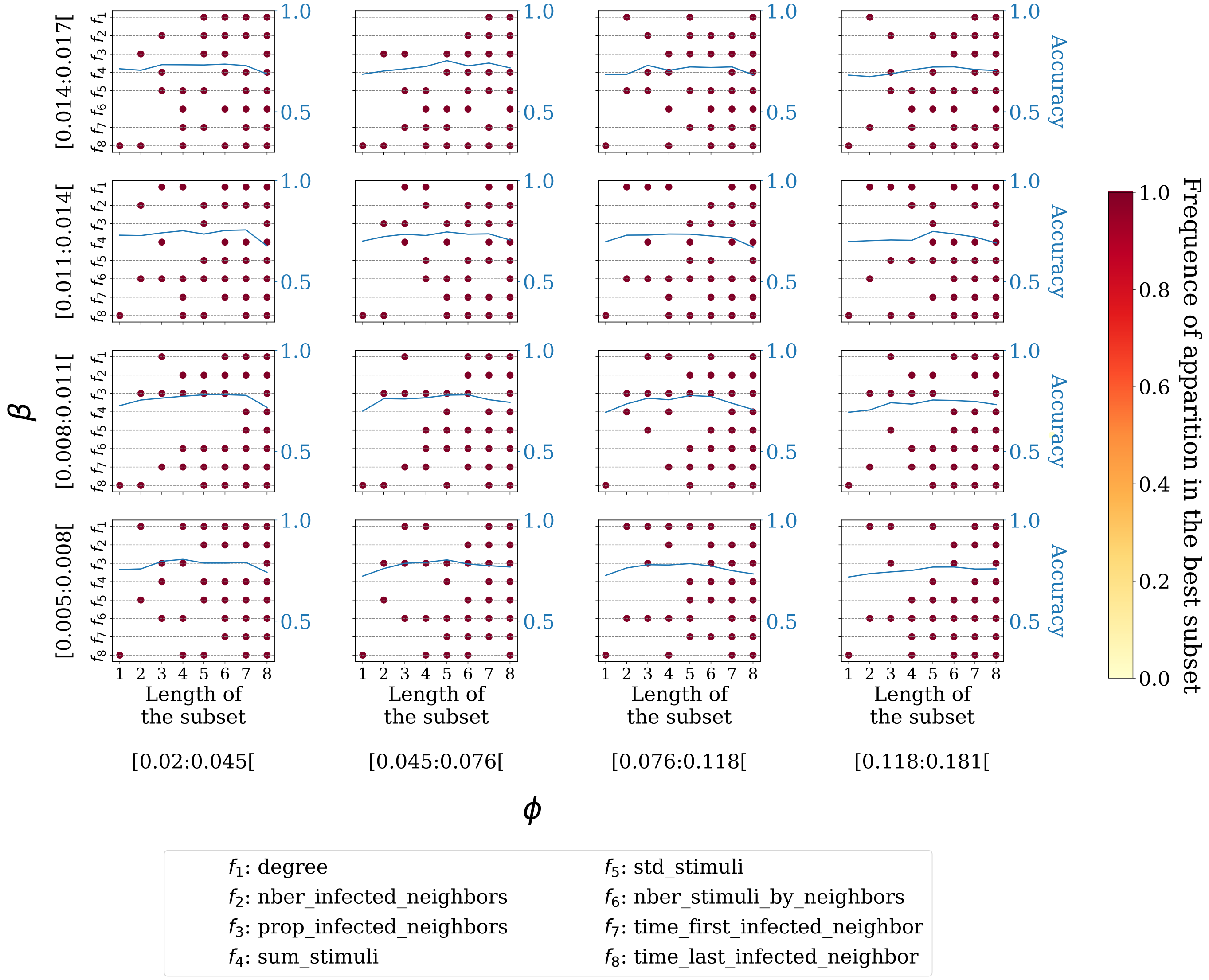

Figure S4. Subsets of features giving the best accuracies (y-axis of each subplot) in the parameter space (β, ϕ) for a certain length of subset (x-axis of each subplot) in the classification with the random forest of Experiment 3. The corresponding accuracies are displayed in blue. If several subsets give the same best accuracies, we compute the frequency of apparition of each feature in those subsets. In most of the cases, only three features is enough to obtain the same accuracy values than with the total set of features, but those three features are different across the parameter space.

5 Accuracies of the different methods on different networks on Experiment 2

To understand how network structure influences process distinguishability, we apply the classification methods on Experiment 2 and 3 on various networks (Figure S5). The values of accuracies remain consistent across the Barabási-Albert, Watts Strogatz and stochastic block model networks. However, we observe a decrease of 0.02 on the accuracy average considering a true Twitter network, but with the machine learning method with unknown parameters. Indeed, one of the most important feature of this method is the degree (Figure 3), which present larger variation with the Twitter network.

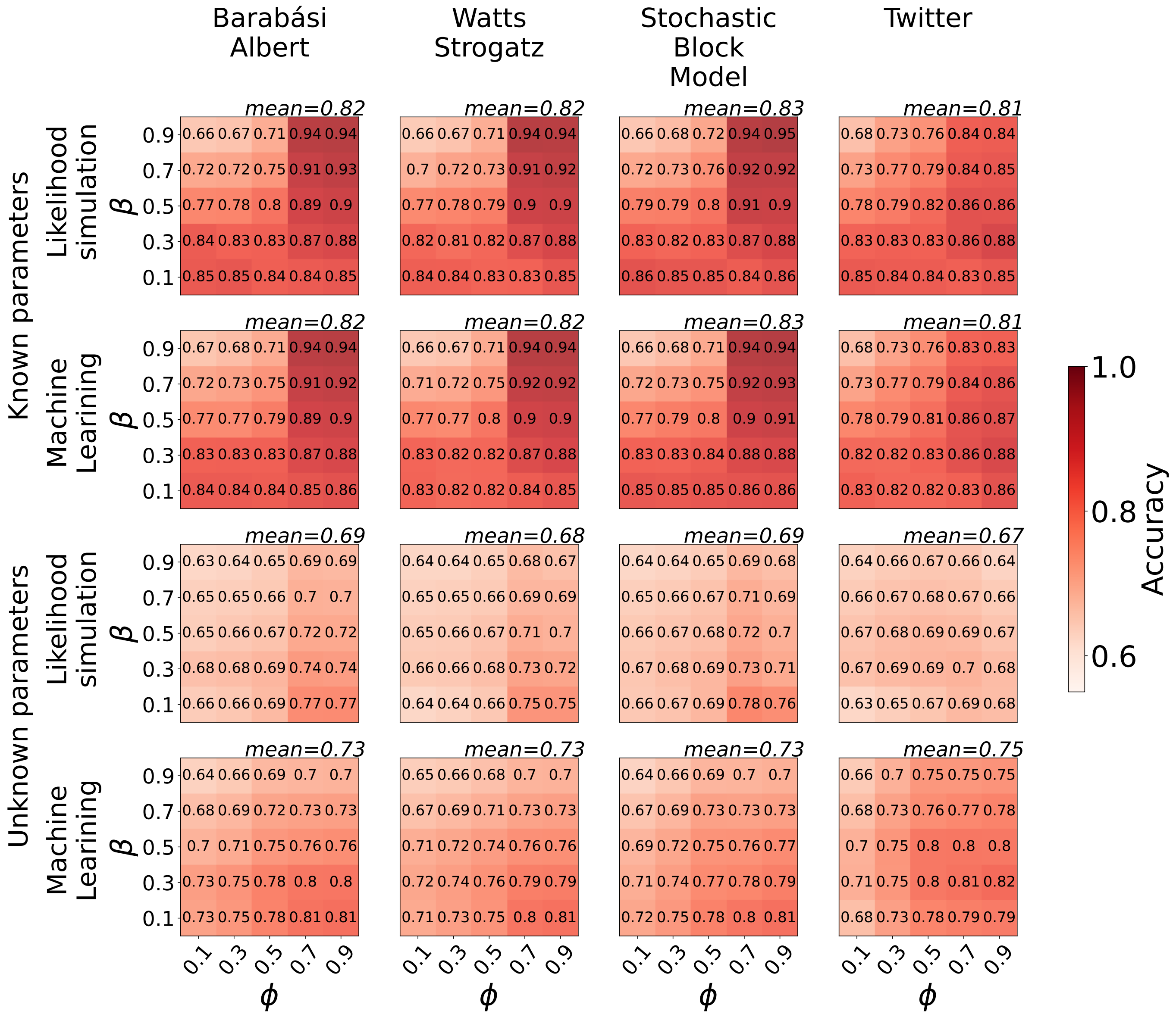

Figure S5. Accuracies obtained by classifying the infection instances from Experiment 2 and 3 on different networks (x-axis) and with different methods (y-axis). The values of accuracies do not depend on the structure of the network.

6 Accuracies of the classification of spontaneous adoption on Experiment 3

We assess the classification accuracies of the simple, complex and spontaneous cases from Experiment 4 with the random forest algorithm, using different values of filtering on the values of $\hat{\beta}$ and $\hat{\phi}$ (40%, 60%, 80% and 100%). The accuracies of the classification of the simple and complex instances increase while the percentage of the filtering diminishes (Figure S6), even though all the obtained values remain above the accuracy of the random classification (0.33). We choose to work with a filter of 80% which presents accuracies above 0.65 while keeping most of the values of the distribution. The accuracies of the classification of the spontaneous instances (Table S2) are consistently low, regardless the percentage of data filtering employed in Experiment 3. This is attributed to the inability to assess the rate of infection r.

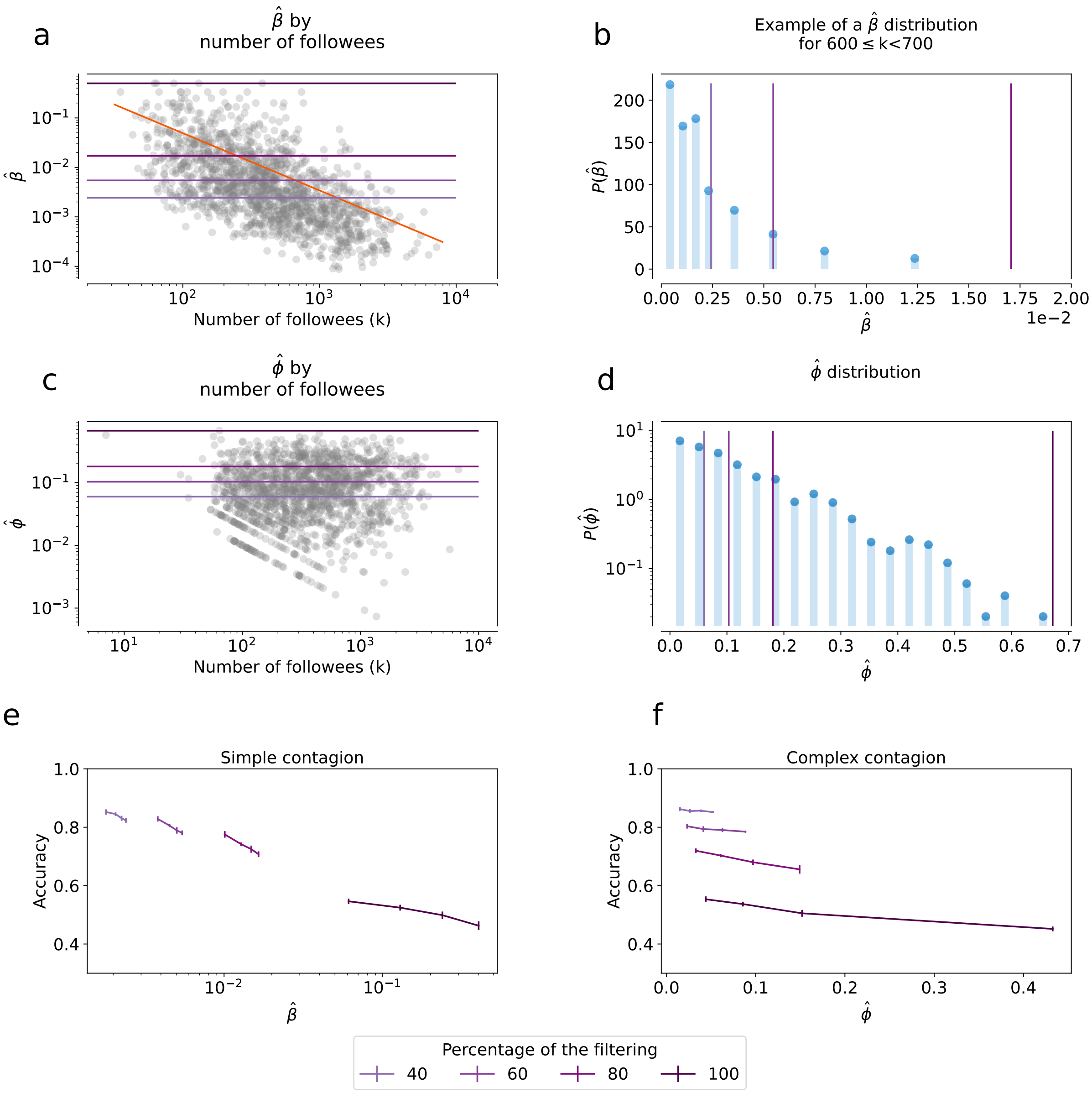

Figure S6. Parametrization and accuracy of the classification of Experiment 4 constructed based on the #GiletsJaunes Twitter dataset. The distributions of $\hat{\beta}$ and $\hat{\phi}$ respectively panels a-b and c-d are filtered keeping their 40%, 60%, 80% or 100% lower values. The accuracy values of the classification of the simple contagion (panel e) and the complex contagion (panel f) increase while the percentage of filtering increases.

Percentage of filtering	40	60	80	100
Accuracy of the St	0.07	0.12	0.23	0.50

Table S2. Accuracy of the classification of the spontaneous adoptions on the Experiment 4 with the random forest

References

1. Zhang, H. The optimality of naive bayes. *Aa* **1**, 3 (2004).

2. Mucherino, A. *et al.* K-nearest neighbor classification. *Data mining agriculture* 83–106 (2009).

3. Gallant, S. I. *et al.* Perceptron-based learning algorithms. *IEEE Transactions on neural networks* **1**, 179–191 (1990).

4. Hsu, C.-W., Chang, C.-C., Lin, C.-J. *et al.* A practical guide to support vector classification (2003).

5. Song, Y.-Y. & Ying, L. Decision tree methods: applications for classification and prediction. *Shanghai archives psychiatry* **27**, 130 (2015).

6. Breiman, L. Random forests. *Mach. learning* **45**, 5–32 (2001).

7. Wang, R. Adaboost for feature selection, classification and its relation with svm, a review. *Phys. Procedia* **25**, 800–807 (2012).

8. Natekin, A. & Knoll, A. Gradient boosting machines, a tutorial. *Front. neurorobotics* **7**, 21 (2013).
